# Relationship between Apparent Diffusion Coefficient Distribution and Cancer Grade in Prostate Cancer and Benign Prostatic Hyperplasia

**DOI:** 10.3390/diagnostics12020525

**Published:** 2022-02-18

**Authors:** Shigeyoshi Saito, Yoshihiro Koyama, Junpei Ueda, Takashi Hashido

**Affiliations:** 1 Department of Medical Physics and Engineering, Division of Health Sciences, Osaka University Graduate School of Medicine, Osaka 565-0871, Japan; uedaj@sahs.med.osaka-u.ac.jp; 2 Department of Advanced Medical Technologies, National Cerebral and Cardiovascular Center Research Institute, Osaka 565-0871, Japan; 3 Department of Medical Technology, Division of Radiology, Osaka University Hospital, Osaka 564-8565, Japan; koyamayo@hp-rad.med.osaka-u.ac.jp (Y.K.); t_hashido@hp-rad.med.osaka-u.ac.jp (T.H.)

**Keywords:** histogram-derived ADC parameters, stromal hyperplasia, glandular hyperplasia, prostate cancer, benign prostate hyperplasia

## Abstract

The aim of this paper was to assess the associations between prostate cancer aggressiveness and histogram-derived apparent diffusion coefficient (ADC) parameters and determine which ADC parameters may help distinguish among stromal hyperplasia (SH), glandular hyperplasia (GH), and low-grade, intermediate-grade, and high-grade prostate cancers. The mean, median, minimum, maximum, and 10th and 25th percentile ADC values were determined from the ADC histogram and compared among two benign prostate hyperplasia (BPH) groups and three Gleason score (GS) groups. Seventy lesions were identified in 58 patients who had undergone proctectomy. Thirty-nine lesions were prostate cancers (GS 6 = 7 lesions, GS 7 = 19 lesions, GS 8 = 11 lesions, GS 9 = 2 lesions), and thirty-one lesions were BPH (SH = 15 lesions, GH = 16 lesions). There were statistically significant differences in 10th percentile and 25th percentile ADC values when comparing GS 6 to GS 7 (*p* < 0.05). The 10th percentile ADC values yielded the highest area under the curve (AUC). Tenth and 25th percentile ADCs can be used to more accurately differentiate lesions with GS 6 from those with GS 7 than other ADC parameters. Our data indicate that the major challenge with ADC mapping is to differentiate between SH and GS 6, and SH and GS 7.

## 1. Introduction

The Gleason score (GS) is an important determinant of the biological activity and aggressiveness of prostate cancer. The risk of prostate cancer progression is a determinant of its management. Prostate cancer is stratified into three risk categories that estimate the probability of disease progression. These categories are low risk (GS = 6 tumors), intermediate-risk (GS = 7 tumors), and high risk (GS ≥ 8 tumors) [1,2]. Benign prostatic hyperplasia (BPH) can mimic prostate cancer symptoms. However, diagnosis would help rule cancer out in cases of BPH, which is due to a combination of stromal and glandular hyperplasia, predominantly of the transitional zone (TZ) [3]. Glandular tissue that is hyperintense on T_2_-weighted (T_2_W) images and stromal tissue that is hypointense can mimic prostate cancer [4,5]. Early differential diagnosis between prostate cancer and BPH is important because both the outcomes of and treatments for these two prostatic diseases are different.

Diffusion-weighted imaging (DWI) quantifies random Brownian motion properties of water molecules in tissues and cells. Restrictions on water diffusion in tissue and cells is inversely correlated to tissue cellularity and cell membrane integrity [6]. Diffusion of molecules occurs across tissues from areas of restricted diffusion to areas with free diffusion. Apparent diffusion coefficient (ADC) is the net displacement of molecules across an area of tissue per second (mm^2^/s). DWI and ADC maps obtained with magnetic resonance imaging (MRI) provide information regarding cellularity and grades of various tumors [7,8,9]. DWI is also used for tumor localization within the prostate [10,11,12]. In addition, ADC measurements derived from DWI are inversely correlated with GS [13,14,15]. The mean ADC is a commonly used ADC measurement because of its relative simplicity [16]. However, some studies have used other parameters, such as median ADC [17], or the 10th [18] or 25th [19] percentile values of the ADC histogram. It is important to clarify which of the ADC-derived parameters reflect tumor aggressiveness and can help distinguish between BPH and prostate cancer.

The present study aimed to assess the associations between prostate cancer aggressiveness as evaluated by GS and histogram-derived ADC parameters. We also aimed to determine which histogram ADC parameters may help distinguish between stromal hyperplasia (SH), glandular hyperplasia (GH), and low-grade, intermediate-grade, and high-grade prostate cancer lesions.

## 2. Materials and Methods

### 2.1. Patients

The local ethics committee approved the use of the clinical data for research and waived the requirement for written informed consent from the patients. Fifty-eight patients who had undergone 12-core and target biopsy of prostate (70 lesions) within 3 months after MRI study were included in this retrospective study; their characteristics are listed in Table 1. After prostatectomy, the prostatic specimens were submitted for histological investigation. The pathologists at our institution outlined each lesion on the microscopic slices and assigned a Gleason score. Thirty-nine lesions were prostate cancers (GS 6 = 7 lesions, GS 7 = 19 lesions, GS 8 = 11 lesions, GS 9 = 2 lesions), and thirty-one lesions were BPH (SH = 15 lesions, GH = 16 lesions).

### 2.2. Magnetic Resonance Imaging

**Figure 1:**
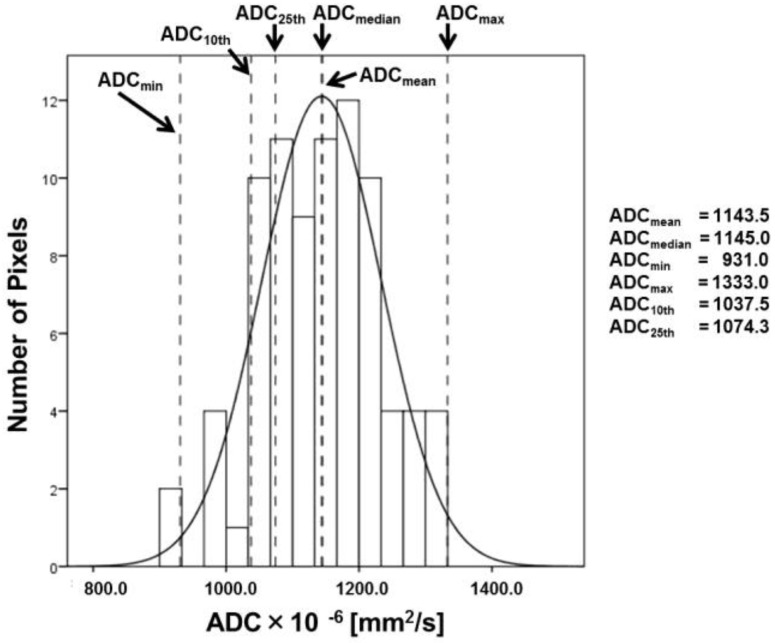
Typical histogram analysis of prostate cancer (Gleason score = 7) using the six histogram-derived ADC parameters. The mean (1143.5 × 10^−6^ mm^2^/s), median (1145.0 × 10^−6^ mm^2^/s), minimum (931.0 × 10^−6^ mm^2^/s), maximum (1333.1 × 10^−6^ mm^2^/s), and 10th (1037.5 × 10^−6^ mm^2^/s) and 25th (1074.3 × 10^−6^ mm^2^/s) percentile ADC values were determined from the ADC histogram.

All MRI data were obtained using a 3.0 T MR scanner (Discovery MR750, GE Healthcare, Milwaukee, WI, USA) with a 32-channel body array coil with eight anterior and eight posterior elements. The sequences included fast spin echo (FSE) T_2_W imaging and DWI. FSE T_2_W imaging was performed with the following parameters: repetition time (TR)/echo time (TE) = 8000/80 ms; matrix = 512 × 384; field of view (FOV) = 24 cm; section thickness = 4 mm with 0.4-mm intersection gap, 25 sections; number of excitations (NEX) = 2; array spatial sensitivity encoding technique (ASSET, GE Healthcare) factor = 1.5 and bandwidth = ±41.7 kHz. DWI was performed with the following parameters: TR/TE = 7500/56.5 ms; matrix = 128 × 128; FOV = 30 cm; section thickness = 4 mm with 1.0-mm intersection gap, 26 sections; NEX = 5; bandwidth = ±250 kHz; b-values = 0 and 1000 s/mm^2^; ASSET factor = 2. The ADC maps were reconstructed at a GE workstation. While establishing the size and region for the region of interest (ROI), positioning in the larger area was considered in order to minimize the effect of region on the hemodynamic inhomogeneity of the tumor using T_2_W images and ADC maps. The six histogram-derived ADC parameters (mean ADC, median ADC, minimum ADC, maximum ADC, 10th percentile ADC, and 25th percentile ADC) were calculated by applying these ROIs using IBM SPSS Statistics (Figure 1, Version 23, IBM, Armonk, NY, USA).

### 2.3. Data Analysis

All of the values in all of the groups are expressed as means ± standard deviations (SDs). All analyses were performed using Prism 5 (Version 5, GraphPad Software, Folsom, CA, USA). We used Tukey’s tests for post-hoc analysis following the ANOVAs used to compare the six histogram-derived ADC parameters. Box plots of these ADC parameters were plotted for all lesions. The relationships between the ADC parameters and GS were analyzed in three subgroups (GS 6, GS 7, and GS ≥ 8). We performed nonparametric ROC curve analysis. The area under the ROC curve (AUC) was estimated to evaluate the ability of each ADC parameter to help discriminate tumor aggressiveness (GS 6 vs. GS 7 vs. GS ≥ 8). The ADC parameter with the highest AUC was identified. Spearman correlation coefficients were calculated to establish the between-patient correlation in each group. A *p* value of <0.05 was considered significant.

## 3. Results

### 3.1. Patients

In total, 70 tumor foci from 58 patients with confirmed prostatectomy were histologically detected. Of these lesions, 20 originated in the peripheral zone (PZ), 46 originated in the TZ, and the remaining 4 lesions covered both the PZ and the TZ. The clinical characteristics of the patients and those of the tumor foci ROIs are summarized in Table 1.

### 3.2. ADC Parameters of All Lesions

**Figure 2:**
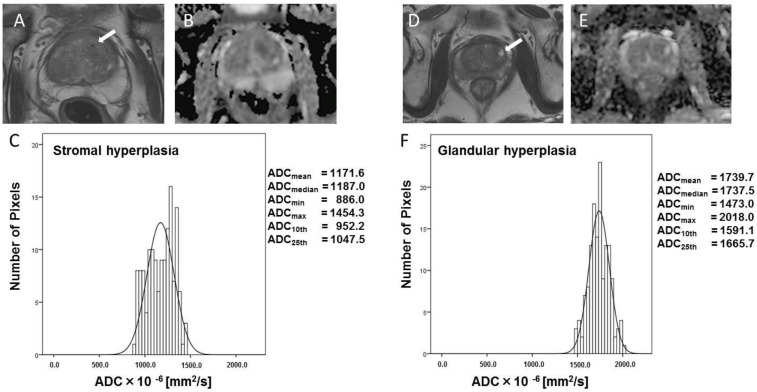
Typical histogram of BPH, and T_2_W and ADC images (stromal hyperplasia and glandular hyperplasia). Typical T_2_W images (**A**,**D**), ADC images (**B**,**E**), and ADC-derived histograms (**C**,**F**) for BPH. The ADC values of the SH foci reflect restricted diffusion in the ADC map ((**B**), ADC_mean_ = 1171.6 × 10^−6^ mm^2^/s). In contrast, the ADC values of the GH foci are seen as bright regions on the ADC map ((**E**), ADC_mean_ = 1739.7 × 10^−6^ mm^2^/s) and do not reflect restricted diffusion. The six histogram-derived ADC parameters are shown for each type of BPH. White arrows indicated BPH.

Figure 2 shows typical T_2_W images (Figure 2A,D), ADC maps (Figure 2B,E), and ADC-derived histograms (Figure 2C,F) of BPH lesions. SH lesions are hypointense (Figure 2A) and GH lesions are hyperintense on T_2_W images (Figure 2D). The ADC parameters of SH foci suggest restricted diffusion in the ADC map (Figure 2B, ADC_mean_ = 1171.6 × 10^−6^ mm^2^/s). However, the ADC values of GH foci are seen as bright regions on the ADC map (Figure 2E, ADC_mean_ = 1739.7 × 10^−6^ mm^2^/s).

**Figure 3:**
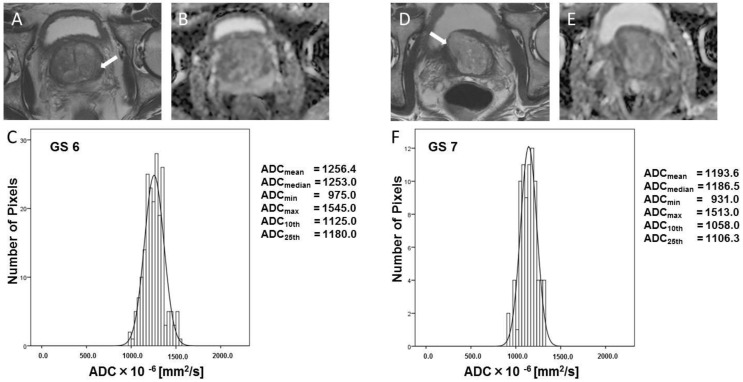
Typical histogram of tumor focus, and T_2_W and ADC images (GS 6 and GS 7). GS = Gleason score. Typical T_2_W images (**A**,**D**), ADC images (**B**,**E**), and ADC-derived histograms (**C**,**F**) for GS 6 and GS 7 prostate cancers. These T_2_W images of prostate cancer illustrate the low signal intensity. The ADC values of the GS 6 lesion reflect slightly restricted diffusion on the ADC map ((**B**), ADC_mean_ = 1256.4 × 10^−6^ mm^2^/s). The ADC values of the GS 7 lesion are lower than that of the GS 6 lesion on the ADC map ((**E**), ADC_mean_ = 1193.6 × 10^−6^ mm^2^/s). White arrows indicated tumor foci.

**Figure 4:**
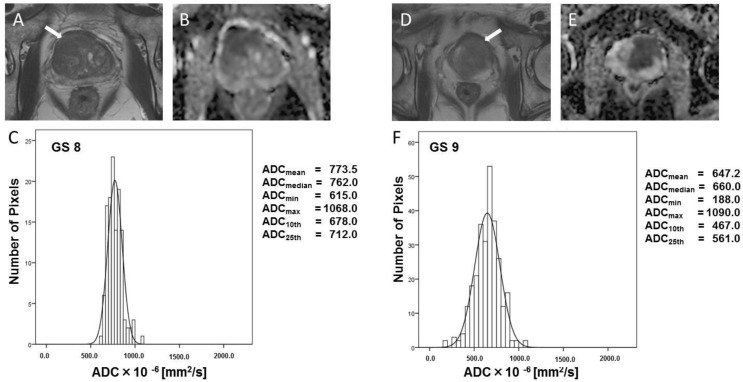
Typical histogram of tumor focus, and T_2_W and ADC images (GS 8 and GS 9). GS = Gleason score. Typical T_2_W images (**A**,**D**), ADC images (**B**,**E**), and ADC-derived histogram (**C**,**F**) for GS 8 and GS 9 prostate cancers. These T_2_W images of prostate cancer illustrate the low signal intensity. The ADC values of the GS 8 ((**B**), ADC_mean_ = 773.5 × 10^−6^ mm^2^/s) and GS 9 ((**D**), ADC_mean_ = 647.2 × 10^−6^ mm^2^/s) lesions reflect restricted diffusion on the ADC map. White arrows indicated tumor foci.

Typical T_2_W images, ADC maps, and ADC-derived histograms from prostate cancer lesions as shown in Figure 3 (GS 6 and 7) and Figure 4 (GS 8 and 9). Prostate cancer lesions have low signal intensity on T_2_W images (Figure 3A,D and Figure 4A,D). The ADC values of GS 6 lesions reflect slightly restricted diffusion in the ADC map (Figure 3B, ADC_mean_ = 1256.4 × 10^−6^ mm^2^/s). However, the ADC values of GS 7 lesions are lower than that of GS 6 lesions on the ADC map (Figure 3E, ADC_mean_ = 1193.6 × 10^−6^ mm^2^/s). The ADC values of GS 8 (Figure 4B, ADC_mean_ = 773.5 × 10^−6^ mm^2^/s) and GS 9 (Figure 4D, ADC_mean_ = 647.2 × 10^−6^ mm^2^/s) lesions reflect restricted diffusion on ADC maps.

**Figure 5:**
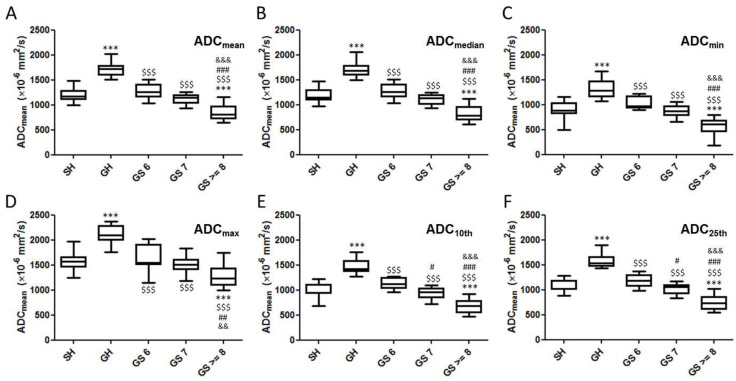
Box plots comparing the six histogram-derived ADC parameters in two BPH groups and the Gleason-graded groups. (**A**) ADC_mean_; (**B**) ADC_median_; (**C**) ADC_min_; (**D**) ADC_max_; (**E**) ADC_10th_; (**F**) ADC_25th_. *: vs. SH (***: *p* < 0.001), $: vs. GH ($$$: *p* < 0.001), #: vs. GS 6 (#: *p* < 0.05, ##: *p* < 0.01, ###: *p* < 0.001), &: vs. GS7 (&&: *p* < 0.01, &&&: *p* < 0.001).

Figure 5 shows box plots of the six histogram-derived ADC for the two BPH and three GS prostate cancer groups. All six ADC parameters differed significantly between SH and GH lesions (Figure 5, *** *p* < 0.001) and SH lesions and GS ≥ 8 tumors (Figure 5, *** *p* < 0.001). All six ADC parameters differed significantly between GH lesions and the three GS prostate cancer groups (Figure 5, $$$ *p* < 0.001). The 10th percentile ADC (Figure 5E, # *p* < 0.05) and 25th percentile ADC (Figure 5F, # *p* < 0.05) were significantly decreased in GS 7 lesions compared to GS 6 lesions. However, four other parameters (mean ADC, median ADC, minimum ADC, and maximum ADC) were not significantly different between GS 7 lesions and GS 6 lesions. All six ADC parameters differed significantly between the GS 6 and GS ≥ 8 prostate cancer groups (Figure 5, ### *p* < 0.001). In addition, all six ADC parameters differed significantly between GS 7 and GS ≥ 8 tumors (Figure 5, &&& *p* < 0.001). There were no significant differences in histogram-derived ADC parameters between SH and GS 6 lesions, and between the SH and GS 7 lesions.

### 3.3. Ability of ADC Parameters to Help Differentiate among Tumors with Different GS

When differentiating between SH and GH lesions, mean, median, 10th percentile, and 25th percentile ADC values yielded the highest AUCs (1.00, *p* < 0.0001, Table 2), while minimum and maximum ADC values yielded the lowest AUCs (0.98, *p* < 0.0001, Table 2). When comparing SH lesions to GS 6 lesions, the AUC of the 10th percentile ADC value (0.82, *p* = 0.02, Table 2) was higher than that of the other parameters. When comparing SH lesions to GS 7 lesions, the AUCs of the six ADC parameters were not significantly different. When comparing the GH group to the three GS prostate cancer groups, the AUCs of the six ADC parameters were high (Table 3). When differentiating tumor foci with GS 6 from those with GS 7, the 10th percentile ADC yielded the highest AUC (0.87, *p* < 0.001, Table 4), while the maximum ADC yielded the lowest AUC (0.67, *p* = 0.20, Table 4). However, the AUC of the 10th percentile ADC was significantly higher than that of the mean ADC.

### 3.4. Between-Subject Spearman Correlation Coefficients for Correlation of ADC Parameters with Gleason Score

Of the six ADC parameters, 10th percentile ADC correlated most strongly with GS (r = −0.84, *p* < 0.001), followed by the 25th percentile ADC (r = −0.82, *p* < 0.001), minimum ADC (r = −0.83, *p* < 0.001), median ADC (r = −0.82, *p* < 0.001), and mean ADC (r = −0.80, *p* < 0.001) (Table 5). Maximum ADC correlated most weakly with GS (r = −0.59, *p* < 0.001). These results indicate that the ADC-derived parameters inversely correlate with GS.

## 4. Discussion

We found that the 10th and 25th percentile ADC parameters correlated best with the GS of prostate cancer lesions. In addition, the 10th and 25th percentile ADC parameters performed significantly better than the mean, median, minimum, and maximum ADC parameters in differentiating tumor foci with GS 6 from those with GS 7. The other four parameters (mean, median, minimum, and maximum ADCs) were not significantly different when comparing GS 6 lesions to GS 7 lesions. Thus, the 10th and 25th percentile ADCs were significantly better than the mean, median, minimum, and maximum ADCs when differentiating these two groups. There were no significant differences in ADC parameters between SH and GS 6 lesions, and SH and GS 7 lesions. These data indicate that the major challenge with ADC mapping is to differentiate SH lesions from those due to prostate cancer.

It has been suggested that enlargement of the TZ due to BPH associated with aging can lead to effacement of the central zone and thus poor differentiation between these two zones on MRI. GHs are easily differentiated from prostate cancer in the central gland owing to their predominantly hyperintense T_2_ signal. However, a SH predominantly has hypointense T_2_W characteristics similar to those of prostate cancer in the central gland. SH is significantly more cellular and denser and has less extracellular fluid than GH. These differences may explain the smaller ADCs observed for SH lesions [20]. Our results also indicate that it is not difficult to differentiate between GH and SH, or between GH and prostate cancer, using all six histogram-derived ADC parameters. Recognition of the different histologic subtypes of BPH is important in improving the diagnosis of prostate cancer. The range of Spearman correlation coefficients describing the relationships between ADC parameters and GS lesions in our study (r = −0.59 to −0.84) has a better range than those of previously reported associations between ADC parameters and GS [18,21]. Some overlap was observed between the ADC parameters of prostate cancer lesions of lower GS (GS 6 and GS 7) and those of SH lesions. A previous report shows that there are overlaps between mean ADCs of SH lesions and those of prostate cancer lesions [22]. These findings indicate that a major challenge in ADC mapping is the differentiation between hypointense SH lesions and prostate cancer lesions. 

Conventional MRI is generally considered inadequate for evaluating prostate cancer because of the heterogeneous T_2_ signal intensity in the normal TZ. Consequently, DWI is now incorporated into various cancer detection methods as an imaging biomarker [23]. Although the low ADC values found in most tumors have been attributed to increased cellular density, diffusion can also be influenced by tissue fibrosis, glandular and stromal structures, and tumor shape [24]. The predominant contribution to the DWI signal in the prostate is from extracellular components, such as tubular structures and their fluid content. There is also a lesser contribution from the extracellular stromal space and intracellular components, such as epithelial and stromal cells. In the PZ of the prostate, the abundant self-diffusion of water molecules within the predominant tubular components provide a high signal on ADC maps [25]. In our study, the 10th percentile ADC and 25th percentile ADC were significantly decreased in GS 7 lesions compared to GS 6 lesions. The 10th and 25th percentile ADC values outperform mean, median, minimum, and maximum ADC values when used to differentiate tumors with GS 6 from GS 7 tumors. In addition, the AUC of the 10th percentile ADC was significantly higher than that of the mean ADC in GS 7 lesions compared to GS 6 lesions. The 10th and 25th percentile ADC values outperform mean, median, minimum, and maximum ADC values when used to differentiate tumors with GS 6 from GS 7 tumors. This may be explained by the heterogeneous histological characteristics of prostate cancer foci. A previous report indicates that a large proportion of interspersed normal glandular prostatic tissue may influence mean ADC and median ADC values more than the 10th percentile ADC and 25th percentile ADC, thereby increasing the values of the former parameters [26]. In tumors densely packed with malignant cells, such as GS 8 and GS 9 tumors, the resulting ADC histogram may be less skewed and spread out. Therefore, differences between the evaluated ADC parameters may be less pronounced in these cases. Focal areas of high cellularity are represented to a greater extent by the 10th and 25th percentile ADCs than by the mean, median, minimum, and maximum ADCs within tumors with heterogeneous cellularity, such as those with GS 6 and GS 7. Thus, dispersion of ADC values in low-grade GS 6 and intermediate-grade GS 7 tumors greatly affects the histogram-derived ADC parameters owing to the relativity low population of cancer cells in the ROI analysis.

Our study had several limitations. When comparing the mean ADC to the 10th percentile ADC, the 10th percentile ADC yielded a higher AUC when differentiating prostate cancer from healthy prostatic tissue. However, the two parameters yielded the same AUC when differentiating tumor foci with GS 6 from those with GS 7 in a previous report [18]. This discrepancy between the findings of the previous study and those of our study may be related to the selection of the b-values in the DWI used for ADC determination. Two different sets of b-values were used to acquire diffusion-weighted MR images in the previous study [18], while we used the same b-values in all patients. The conditions of the MR sequences are important when evaluating ADC values in prostate cancer. It is thus important to select and set appropriate b-values. This study was a retrospective study with a relatively small sample size, which may have been influenced by selection and verification biases. Previous reports that Gleason score from needle biopsy can often differ from that determined with radical prostatectomy [27] and with immediate repeat biopsies [28]. The reliability of the correlation between MR imaging and the specimens is crucial. Although we tried to minimize inaccuracies by performing the correlation carefully and avoiding the inclusion of healthy tumor tissue in the ROIs, some uncertainty remains. In addition, we did not normalize the histograms to reduce the effects of tumor volumes. Finally, future studies should evaluate the reproducibility of the absolute ADC values in different vendors and field strengths, as well as the choice of b-values.

## 5. Conclusions

Our results suggest that the 10th and 25th percentile ADCs can be used to more accurately differentiate lesions with GS 6 from those with GS 7 than other ADC parameters, while our data indicate that the major challenge with ADC mapping is to differentiate between SH and GS 6, and SH and GS 7. These parameters can be used as a non-invasive biomarker to predict the Gleason score of the prostate cancer patients.

## Figures and Tables

**Table 1:** Summary of clinical and pathologic characteristics. For clinical characteristics, data are means and standard deviations with ranges in parentheses. For pathologic characteristics, data are numbers of patients with percentages in parentheses. For Gleason grade, data are numbers of tumor foci, with percentages in parentheses.

Variable	Value
Clinical characteristics	
Age (year)	69.3 ± 6.5 (51–84)
PSA (ng/mL)	17.5 ± 25. 7 (4.2–196.0)
Pathologic characteristics	
pT2a	22 (56.4%)
pT2b	1 (2.5%)
pT2c	7 (17.9%)
pT3a	8 (20.5%)
pT4	1 (2.5%)
Gleason grade	
GS 3 + 3	7 (17.9%)
GS 3 + 4	17 (43.6%)
GS 4 + 3	2 (5.1%)
GS ≥ 8	13 (33.3%)

**Table 2:** Comparison of AUC values for used to differentiate tumor foci from stromal hyperplasia.

	SH vs. GS 6	*p* Value	SH vs. GS 7	*p* Value	SH vs. GS 8	*p* Value	SH vs. GH	*p* Value
Mean	0.70	0.15	0.62	0.27	0.97	<0.0001	1.00	<0.0001
Median	0.69	0.17	0.63	0.24	0.98	<0.0001	1.00	<0.0001
Minimum	0.76	0.05	0.59	0.41	0.96	<0.0001	0.98	<0.0001
Maximum	0.50	0.97	0.66	0.13	0.88	0.0003	0.98	<0.0001
10th	0.82	0.02	0.56	0.54	0.97	<0.0001	1.00	<0.0001
25th	0.73	0.08	0.59	0.41	0.97	<0.0001	1.00	<0.0001

**Table 3:** Comparison of AUC values used to differentiating tumor foci from glandular hyperplasia.

	GH vs. GS 6	*p* Value	GH vs. GS 7	*p* Value	GH vs. GS 8	*p* Value
Mean	1.00	<0.0001	1.0	<0.0001	1.0	<0.0001
Median	0.99	0.0002	1.0	<0.0001	1.0	<0.0001
Minimum	0.91	0.002	1.0	<0.0001	1.0	<0.0001
Maximum	0.94	0.001	1.0	<0.0001	1.0	<0.0001
10th	1.00	0.0002	1.0	<0.0001	1.0	<0.0001
25th	1.00	0.0002	1.0	<0.0001	1.0	<0.0001

**Table 4:** Comparison of AUC values used to differentiate tumor foci from prostate cancer.

	GS 6 vs. GS 7	*p* Value	GS 6 vs. GS 8	*p* Value	GS 7 vs. GS 8	*p* Value
Mean	0.78	0.04	0.98	0.0003	0.94	<0.0001
Median	0.79	0.03	0.99	0.0002	0.95	<0.0001
Minimum	0.79	0.03	1.00	0.0002	0.95	<0.0001
Maximum	0.67	0.20	0.86	0.008	0.83	0.002
10th	0.87	0.01	1.00	0.0002	0.94	<0.0001
25th	0.79	0.03	0.99	0.0002	0.95	<0.0001

**Table 5:** Between-subjects Spearman correlation coefficients for correlations between ADC parameters and Gleason score.

	Spearman r	*p* Value
Mean	−0.80	<0.0001
Median	−0.82	<0.0001
Minimum	−0.83	<0.0001
Maximum	−0.59	<0.0001
10th	−0.84	<0.0001
25th	−0.82	<0.0001

## Data Availability

Not applicable.
